# Enhancing eye tracking for nonhuman primates and other subjects unable to follow instructions: Adaptive calibration and validation of Tobii eye trackers with the Titta toolbox

**DOI:** 10.3758/s13428-024-02540-y

**Published:** 2024-12-04

**Authors:** Diederick C. Niehorster, Will Whitham, Benjamin R. Lake, Steven J. Schapiro, Ian M. Andolina, Jessica L. Yorzinski

**Affiliations:** 1 Lund University Humanities Lab, Lund University, Box 201, 221 00 Lund, Sweden; 2 Department of Psychology, Lund University, Lund, Sweden; 3 Department of Psychology and Special Education, Texas A&M University - Commerce, Commerce, TX USA; 4 Ecology and Evolutionary Biology Program, Texas A&M University, College Station, TX USA; 5 Department of Comparative Medicine, UT MD Anderson Cancer Center, Bastrop, TX USA; 6 Institute of Neuroscience, State Key Laboratory of Neuroscience, Key Laboratory of Primate Neurobiology, Center for Excellence in Brain Science and Intelligence Technology, Chinese Academy of Sciences, Shanghai, 200031 China; 7 Department of Ecology and Conservation Biology, Texas A&M University, College Station, TX USA

**Keywords:** Apes, Calibration, Equipment interface, Eye tracking, Eye movements, Infants, Monkeys, Primates, Tobii, Validation

## Abstract

Accurate eye tracking is crucial for gaze-dependent research, but calibrating eye trackers in subjects who cannot follow instructions, such as human infants and nonhuman primates, presents a challenge. Traditional calibration methods rely on verbal instructions, which are ineffective for these populations. To address this, researchers often use attention-grabbing stimuli in known locations; however, existing software for video-based calibration is often proprietary and inflexible. We introduce an extension to the open-source toolbox Titta—a software package integrating desktop Tobii eye trackers with PsychToolbox experiments—to facilitate custom video-based calibration. This toolbox extension offers a flexible platform for attracting attention, calibrating using flexible point selection, and validating the calibration. The toolbox has been refined through extensive use with chimpanzees, baboons, and macaques, demonstrating its effectiveness across species. Our adaptive calibration and validation procedures provide a standardized method for achieving more accurate gaze tracking, enhancing gaze accuracy across diverse species.

**Supplementary information:**

The online version contains supplementary material available at 10.3758/s13428-024-02540-y.

## Introduction

The calibration of eye trackers is a crucial step in accurately and precisely monitoring gaze behavior (Holmqvist et al., 2011). Calibrating healthy human adults is relatively simple: experimenters ask the participants to fixate on visual targets (e.g., dots) in known locations. Eye tracker algorithms can then quantify the difference (i.e., minimize the error) between participants’ known gaze position (the target locations) and the gaze coordinate information recorded by the tracker (Aslin & McMurray, 2004). Calibration critically relies on the assumption that participants look at the presented calibration targets. This makes calibration much more difficult in participants that are unable or unwilling to follow verbal instructions, such as infants (Schlegelmilch & Wertz, 2019), children with autism spectrum disorder (Sasson & Elison 2012), and nonhuman animals (Hopper et al., 2021; Park et al., 2023; Yorzinski et al., 2013). Poor calibrations often result in participants being excluded from experiments (Haith, 2004).

Various methods have been used to calibrate participants that do not follow verbal instructions. One method is to use an oculometric approach based on corneal reflections (Fantz, 1958; Hamada, 1984). This method relies on reflections of objects off the corneal surface to establish several calibration points (Maurer, 1975; Hamada, 1984; Yorzinski et al., 2013; see also Nitschke & Nakazawa, 2017). Another method for getting participants to look at known locations is a dimming-detection task (Wurtz, 1969; Foeller & Tychsen 2002). This task involves training participants to release a lever when a spot on a display monitor dims. Because the spot is very small, the participants need to fixate on the spot in order to detect the dimming. While mature nonhuman primates can be trained with positive reinforcement alongside food or fluid control to complete a calibration task, such careful behavioral shaping is not always possible. For instance, time is often limited when working in a zoo, or with young or disease model participants, in which case methods that both minimize training requirements and improve calibration flexibility are preferred. This is one of the reasons that a common calibration method involves naturally guiding the attention of participants to the right locations using attracting stimuli. These stimuli may, for instance, consist of food (e.g., Williams et al., 2011), toys (e.g., Bradshaw et al., 2023), or videos (e.g., Kano et al., 2012; Leppänen et al., 2022; Schlegelmilch & Wertz, 2019). When calibrating eye trackers using video stimuli, experimenters often use commercial or custom software that is not freely available or readily accessible for use in other studies. In addition, this software often has limited flexibility in adjusting the calibration procedure (Zeng et al., 2024). Given the greater individual and day-to-day variation in behavioral performance of young participants and other participants who are unable or unwilling to follow verbal instructions, the lack of adaptability in the calibration procedure increases frustration for the experimenter and can lead to participant noncompliance. Furthermore, this software varies in how validations are performed (Hopper et al., 2021), making it difficult to compare eye tracker accuracy across studies.

We therefore developed an open-source toolbox implementing a flexible and adaptive interface for calibrating and validating eye trackers using videos or other attention attracting stimuli. Our toolbox is an extension of Titta (Niehorster et al., 2020a), a software package that integrates desktop Tobii eye trackers with experiments written in MATLAB with PsychToolbox (Brainard, 1997; Kleiner et al., 2007; Pelli, 1997) and is provided as part of the Titta distribution at https://github.com/dcnieho/titta. While the core Titta functionality is also available for Python and PsychoPy (Peirce, 2007, 2009), our extension is only available for MATLAB. Besides a flexible calibration interface that can be used with any participant who is unable to follow instructions such as human infants and nonhuman primates, the current contribution also includes a procedure for automatically calibrating nonhuman primates. This procedure consists of three phases: attention-grabbing, calibration, and validation. During the attention-grabbing phase, full-screen videos are played to attract the participant to look at the display monitor. These videos progressively shrink until they are the size of the calibration videos. This approach of progressively shrinking stimuli is common in touchscreen and joystick designs with primates (Calapai et al., 2022; Evans et al., 2008; Washburn & Rumbaugh, 1992). During the calibration phase, a small video is played at specific locations on the display monitor until calibration is completed (a minimum of two calibration locations is needed for our procedure). Calibration can be triggered manually or automatically, depending on the experimenter’s preference. Importantly, calibration is iterative and flexible, points can be added until calibration is achieved, and individual points can be recollected. Lastly, the validation phase is performed automatically by playing small videos in locations spread in a grid along the display monitor and collecting validation data when the participant looks at these videos. At the end of the validation, the accuracy of the calibration is displayed. The eye tracker can be re-calibrated if the accuracy level is deemed unacceptable, and multiple calibration runs can be stored and compared in real time using a selection menu built into the validation interface. Below we detail how the flexible calibration and validation interface as well as the automated procedure for calibration and validation have been implemented and what opportunities for customization exist.

## Implementation

The implementation of our automated calibration and validation procedure for participants who are unable to follow instructions consists of multiple parts. First, a fully featured control panel has been implemented that replaces the standard Titta calibration interface. Among other features, this control panel provides the experimenter with full control over when to show and collect gaze data for specific calibration points, whether and when to re-collect gaze data for calibration points, and which of the collected calibration points are used to compute the calibration. Second, a programming interface has been developed that enables user code to automate the operation of the interface. Instead of the user manually performing the calibration or validation, such as indicating when and for which calibration or validation points to (re-)collect data, the user code can launch such actions. The user code can, for instance, monitor the gaze data provided by the eye tracker in real time and launch collection of calibration data once the gaze is close enough to a given location on the screen and perform calibration once enough data has been collected. Finally, a class has been developed that uses this programming interface to provide an automated calibration and validation procedure for participants who are unable to follow instructions, which was specifically designed for nonhuman primates. Here, we discuss these three parts of the new extensions to the Titta toolbox in turn.

### The advanced calibration/validation interface

**Fig. 1:**
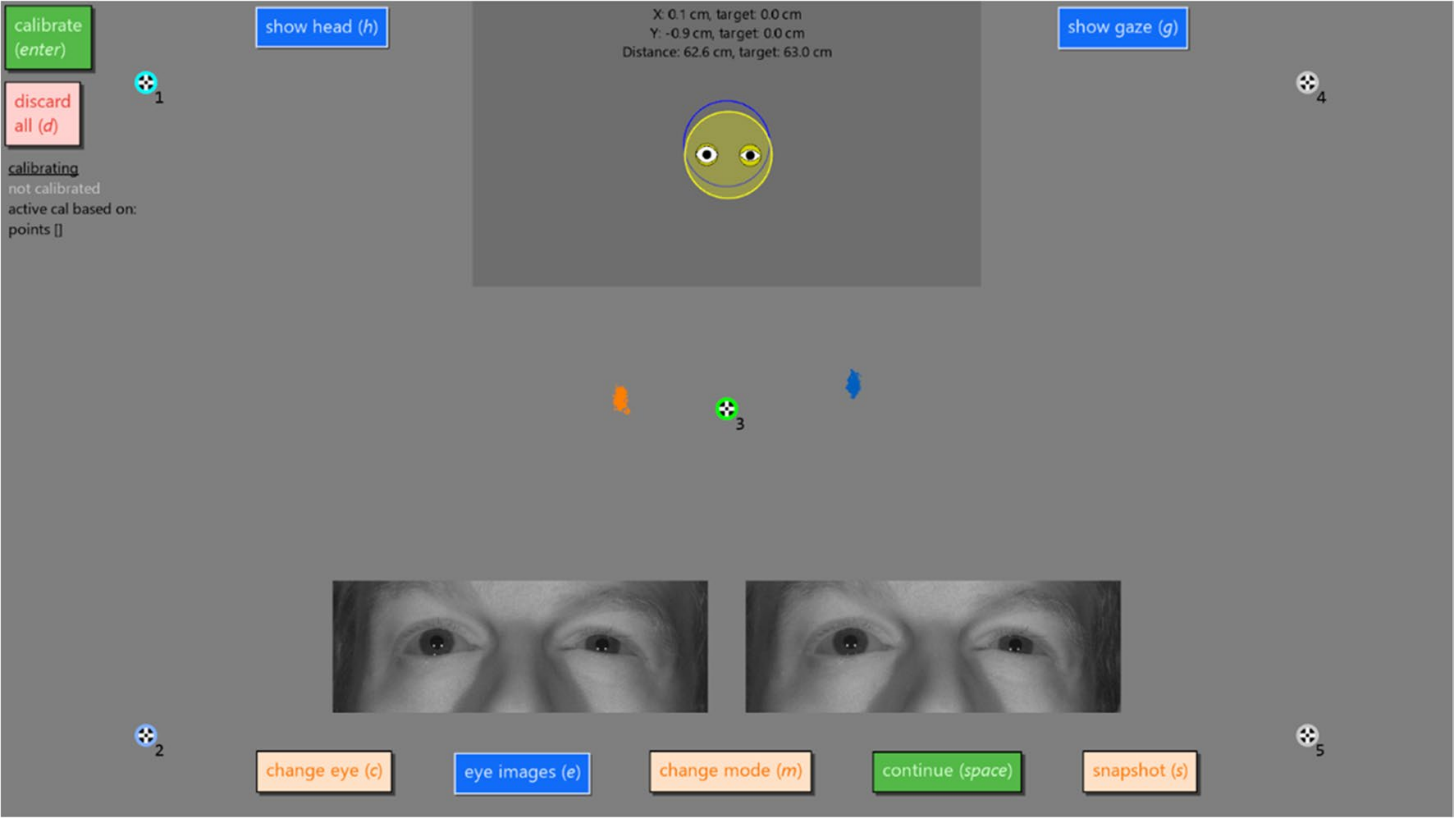
Screenshot of advanced calibration interface. Shown are the five calibration targets. The green annulus around target 3 indicates that calibration data has been collected for this target, the light blue annulus for target 2 that it is being shown to the participant, and the cyan annulus for target 1 that it is enqueued to be shown, while the grey annuli around the other targets indicate that no calibration data has been collected. Further shown are the eye images from the two cameras in a Tobii Pro Spectrum. The head positioning display is shown at the top of the screen, and includes pupils that are sized based on real-time pupil data and eyelids drawn based on eye openness data (note that the right eye is more closed, corresponding to the eye image). The text in the head positioning display indicates the current position of the head in the eye tracker’s user coordinate system, while the target values indicate the configured desired position (indicated graphically by the blue circle). Finally shown are the recorded gaze positions for the last 500 ms for the left (cloud of small orange points) and right (blue points) eyes. The large offset from where the participant was looking (the center target) is because the eye tracker is not calibrated, as indicated by the status information (“not calibrated” in grey) on the left of the screen. Keyboard shortcuts for operating the interface in lieu of using the mouse are indicated in parentheses

Figure 1 shows a screenshot of the interface for calibrating desktop Tobii eye trackers that is displayed when calling the Titta.calibrateAdvanced() function. We will discuss the functionality of this advanced calibration interface using this screenshot.

Upon entering the calibration interface, by default the eye tracker will be in an uncalibrated state.^1^ In the interface, a set of numbered fixation targets are seen spread across the screen. These are calibration targets that can be enqueued to be shown to the participant for calibration data collection by clicking them with the mouse or pressing their corresponding number on the keyboard. This is different from the previously implemented Titta.calibrate() function (Niehorster et al., 2020a) for participants who can follow instructions, because there the operator could view but not influence the order and timing of when calibration (or validation) targets are shown. The location and number of potential calibration targets can be configured in the user’s script; the default layout is shown in Fig. 1. The green annulus around target 3 indicates that calibration data has been collected for this target, while the gray annulus around the other calibration targets indicates that no data has been collected for these targets. The collected calibration data for a given target can be removed by holding down the shift key while clicking the target with the mouse or while pressing its corresponding number on the keyboard. All calibration data can be removed by pressing the “discard all” button on the left side of the interface. It is also possible to preload a previous calibration using an optional input argument of the Titta.calibrateAdvanced() function when opening the interface.

Once data for the desired number of calibration points (this does not have to be all calibration points) is collected, the eye tracker can be instructed to perform a calibration using the available data by pressing the “calibrate” button on the left side of the screen. Once the eye tracker reports the result of the calibration, the status information on the left side of the screen is updated. The gray “not calibrated” text is replaced with “calibration succeeded” printed in green if the calibration succeeded (see left side of Fig. 3), or with “calibration failed” printed in red if the calibration was not successful. If the calibration was successful, the interface furthermore indicates which calibration points were used for the active calibration, as reported by the eye tracker.

**Fig. 2:**
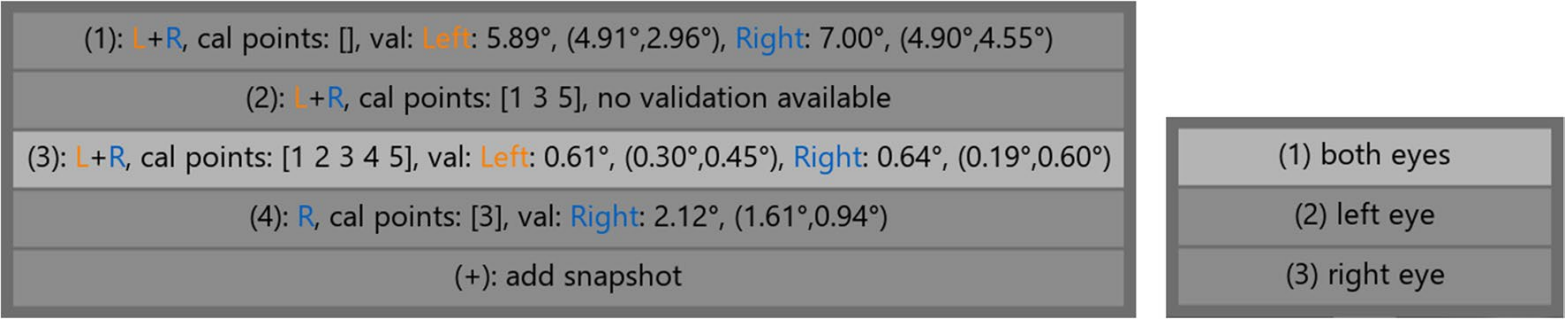
Left: calibration snapshot menu. Shown are (1) a default calibration for both eyes (not based on any collected calibration data); (2) a calibration for both eyes based on three calibration points; (3) a calibration for both eyes based on five calibration points; and (4) a calibration for the right eye based on one calibration point. The third calibration is currently active as indicated by the highlight. The accuracy of the calibrations is shown by means of offsets collected by means of a validation procedure, if available. Right: menu for changing which eye is calibrated

A snapshot of the currently active calibration can be stored using the snapshot button at the bottom of the screen. Such snapshots enable restoring the current calibration state at a later time during the current calibration session. If a previous calibration was loaded using the optional input argument of the Titta.calibrateAdvanced() function, it is also added in the snapshot menu. Having stored snapshots of calibrations means that another calibration can be attempted, but the earlier snapshotted calibration can be reloaded and used for the data recording if the later calibration turns out to be less successful. The left panel of Fig. 2 shows an example of the snapshot menu with multiple such previous snapshots stored and the accuracy of the calibrations in these snapshots as determined through validation (discussed below).

Along the top edge of the screen are two additional buttons, “show head” and “show gaze.” When the show head button is pressed, a movable and resizable overlay is activated that shows Titta’s participant setup display. This display gives information about the participant’s head position and orientation (yellow circle, Fig. 1) and how this relates to a configurable desired position in front of the eye tracker (indicated by a blue circle, Fig. 1). The head display furthermore shows real-time pupil size data and, if the eye tracker supports it, information about eye openness, which is informative about eye blinks (see Nyström et al., 2024). The show gaze button toggles whether a real-time history of gaze positions for a configurable time window (default 500 ms) is shown on the operator screen. For both buttons, the head display and gaze position are only drawn on the operator display by default; the head display and gaze position can also be shown on the participant display by holding down the shift key when activating the respective functions.

Finally, along the bottom side of the screen there are the buttons “change eye,” “eye images,” “continue,” and “change mode.” “Change eye” is available for eye trackers that offer monocular calibration functionality (e.g., the Tobii Pro Spectrum) and when pressed brings up a menu that allows selecting which eye is being calibrated (right panel of Fig. 2). Some models (such as the Tobii Pro Spectrum) provide eye images while others (such as the Tobii Pro TX300) do not. If eye images are provided by the eye tracker, the “eye images” button appears in the interface, which toggles whether the eye images are shown in the interface. The “continue” button is pressed when the operator is done performing a calibration and wants to return control to the caller of the Titta.calibrateAdvanced() function, e.g., to start the data recording. The “change mode” button toggles between calibration and validation mode.

**Fig. 3:**
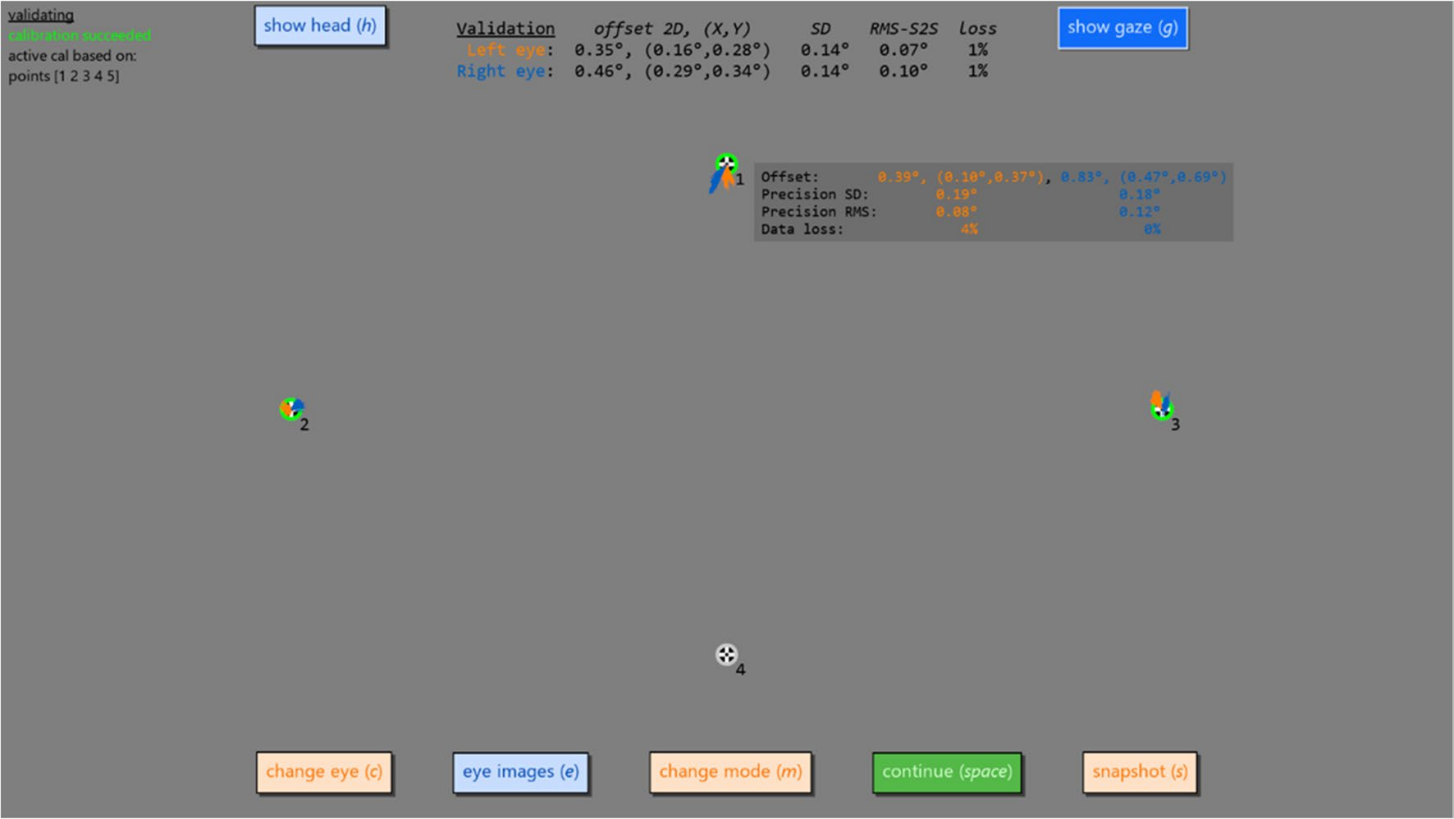
Screenshot of the validation interface. Shown are the four validation targets. The green annuli around three of the targets indicates that validation data has been collected for these targets, while the grey annulus around target 4 indicates that no validation data has been collected. For each validation target, the collected gaze data is visualized by means of orange (left eye) and blue (right eye) lines, one line per sample. At the left of the screen information is shown about the currently active calibration and atop the screen information about the quality of the collected validation data, averaged over the validation points for which data is available. The quality of data for a specific validation target can be shown by hovering the mouse cursor over that point, as is shown for the top point (target 1) in this screenshot

The interface for the validation mode is shown in Fig. 3. On the left side of the screen, the validation interface shows the same information about the activate calibration as the calibration screen, and most of the same buttons as in the calibration interface are available along the edges of the screen. However, on the validation screen, a new configurable set of validation targets is shown, for which data can also be collected or discarded by mouse click or keyboard press. Along the top of the screen, average data quality statistics (Holmqvist et al., 2012; Niehorster et al., 2020b) are shown for the set of collected points. These include the offset (often called accuracy), the root mean square of the sample-to-sample distance (RMS-S2S), and STD measures of precision (see Niehorster et al., 2020c) and data loss. When hovering the mouse over a validation target, the same data quality measures are shown only for the data collected for the hovered validation target.

## Programming interface for automated operation

**Fig. 4:**
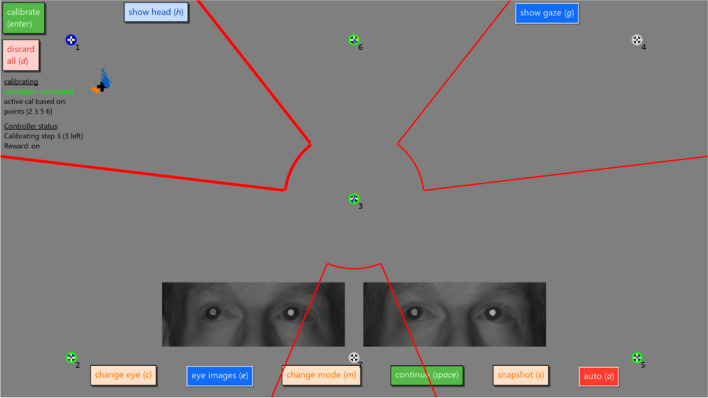
Screenshot of the calibration interface with the example gaze-contingent automated calibration activated (see text). Shown is the state during the third stage of this procedure, where the currently active calibration is based on data collected for four previous calibration points. Further shown is the status message generated by the calibration controller class (left of the image), the arc-shaped AOI delineation (red-outlined areas) used by the example controller to determine what target is looked at. The logic implemented in the example calibration controller is that if its internal gaze representation (black cross) is anywhere within an AOI it is counted as looking at that point and a calibration data collection is triggered. The dark blue annulus around target 1 indicates that calibration data is currently being collected for that target

The advanced calibration interface presented above also has a programming interface for automated operation by a user script. The Titta.calibrateAdvanced() function takes an instance of a calibration controller class as an optional input argument. Such a class enables arbitrary user logic to run during the calibration, to react to calibration events (such as data being collected or a calibration being computed), to provide status text to be shown on the calibration interface screen (see left side of Fig. 4), and to draw on the participant and operator screens. Specifically, the user should implement the following four method functions in a calibration controller class that will be called by the calibration interface:

1. tick(): This function is called every frame (e.g., 60 times per second if the participant display runs at 60 Hz) and can be used to automate the operation of the interface. This method can return commands to the calibration interface, like starting collection of calibration data for a given calibration target, discarding data for a calibration target, or computing a calibration based on the collected data. The user is free to perform any operation during the execution of this method to determine which commands, if any, to issue. The gaze position reported by the eye tracker can, for instance, be monitored and used to trigger, or cancel, collection of calibration data for a calibration target depending on how close to the target the participant is currently looking. The tick() method can also be used for any other logic the user wishes to run, such as providing rewards to the participant based on their gaze position.
2. receiveUpdate(): This function is called to notify the controller class that an event occurred. Events can for instance be the start or completion of data collection for a calibration or validation point, completion of calibration computation, or the user pressing the “auto” button in the interface (Fig. 4) indicating that the automated procedure should start or stop.
3. getStatusText(): This function should return the status text to be shown to the operator on the calibration interface, if any.
4. draw(): Any drawing to the participant or operator screens should be performed from this function. One may, for instance, wish to play a video to the participant to retain attention when no calibration or validation targets are being shown, or annotate the operator screen with an area of interest (AOI) delineation that is used by the controller to determine which calibration target the participant is currently looking at (cf. Figure 4). One can also draw the current internal representation of participant gaze used by the controller, since this may be different from the real-time gaze display that can be toggled in the calibration interface if, for instance, incoming gaze data is filtered by a temporal averaging procedure.

When a calibration controller class is registered with the interface, the “auto” button appears in the row of buttons at the bottom of the calibration and validation interface (Fig. 4). When the calibration interface loads, the user’s calibration controller will not be activated yet. The operator starts the automated procedure by pressing the “auto” button, and can halt it and take over themselves at any time by clicking the button again. Activating or deactivating the auto mode sends a notification to the calibration controller signaling it to start or stop its operation. It should be noted, however, that the controller’s method functions are called regardless of whether auto mode is activated. This enables, for instance, the provision of rewards to retain participant engagement before the automated procedure starts, during, or after it completes.

As an example, to demonstrate the functionality, Titta comes with an automated calibration controller designed for human adults that gaze-contingently determines which calibration point to collect data for (located at demo_experiments/ readmeAdvancedCalibration_auto.m in the GitHub repository). When activated, it starts by showing the participant a single calibration target in the center of the screen. When gaze is close enough to this point, the controller triggers the collection of calibration data (c.f. Leppänen et al., 2022) and, once notified that the data was collected, triggers computation of a calibration based on this single calibration point. Once successfully calibrated, three new points are shown at the same time to the participant. An AOI delineation (see Fig. 4) is used to determine to which, if any, calibration target the participant is looking closely enough for a calibration data collection to be started for that point. Once calibration data is collected for all three calibration targets, a new calibration is computed, and a third stage with a further three points is entered following the same procedure. This third stage is shown in Fig. 4.

It should be noted that this controller programming interface can also be used for much simpler purposes than to implement a completely automated calibration and validation procedure. For instance, the class methods could be used simply to monitor whether the participant gazes at the screen or within a specific area on the screen, provide rewards when they are looking at the correct location, and provide a status text indicating whether rewards are currently being provided or not.

## An automated calibration procedure for nonhuman primates

We have implemented an automated procedure to calibrate participants who do not follow verbal instructions, aimed at nonhuman primates. Like the other automated procedure described in the previous section, this procedure is implemented as an automated calibration controller. The parameters mentioned in the description of the procedure below are default settings, but all can be configured. We have tested this automated procedure and obtained successful calibrations in captive adult chimpanzees (*Pan troglodytes*) and olive baboons (*Papio anubis*) at The University of Texas MD Anderson Cancer Center’s Michale E. Keeling Center for Comparative Medicine and Research, and young cynomolgus macaques (*Macaca fascicularis,* ~ *9 months old*) at the Institute of Neuroscience, Shanghai. At the Keeling Center, we dispensed juice (fruit-flavored, sugar-free drink mix) rewards to the primates using a USB relay (SMAKN LCUS-1) that controlled a peristaltic dosing pump (Gikfun 12 V DC). At the Institute of Neuroscience, we delivered apple juice using an Arduino with a low-latency driver that controlled a Cymoer peristaltic pump. Juice can be manually dispensed with a keyboard shortcut (“j”). The automatic procedure dispensed juice only when the participants were looking at videos shown by the procedure (no juice was dispensed if the participants were looking onscreen at locations other than the video).

The automated procedure is implemented in the NonHumanPrimateCalController class and consists of three phases. The first phase that starts running once the calibration controller is activated is designed to grab the attention of participants to the screen of the eye tracker. This is done by playing full-screen videos of interesting scenes to the participants (e.g., videos of other primates in the case of our primate participants) while monitoring the gaze signal recorded by the eye tracker to determine whether the participants are watching the screen. Once attention to the screen has been attracted for sufficiently long (approximately half a minute), the videos shrink progressively (c.f., Washburn & Rumbaugh, 1992; Evans et al., 2008; Calapai et al., 2022; and the infant calibration options in Tobii Pro Lab) as long as they are being watched until they are the size of the calibration videos (300 × 300 pixels). If participants are already well trained to watch the screen and videos on the screen, this first phase can be skipped with a keyboard shortcut (“x”). 

**Fig. 5:**
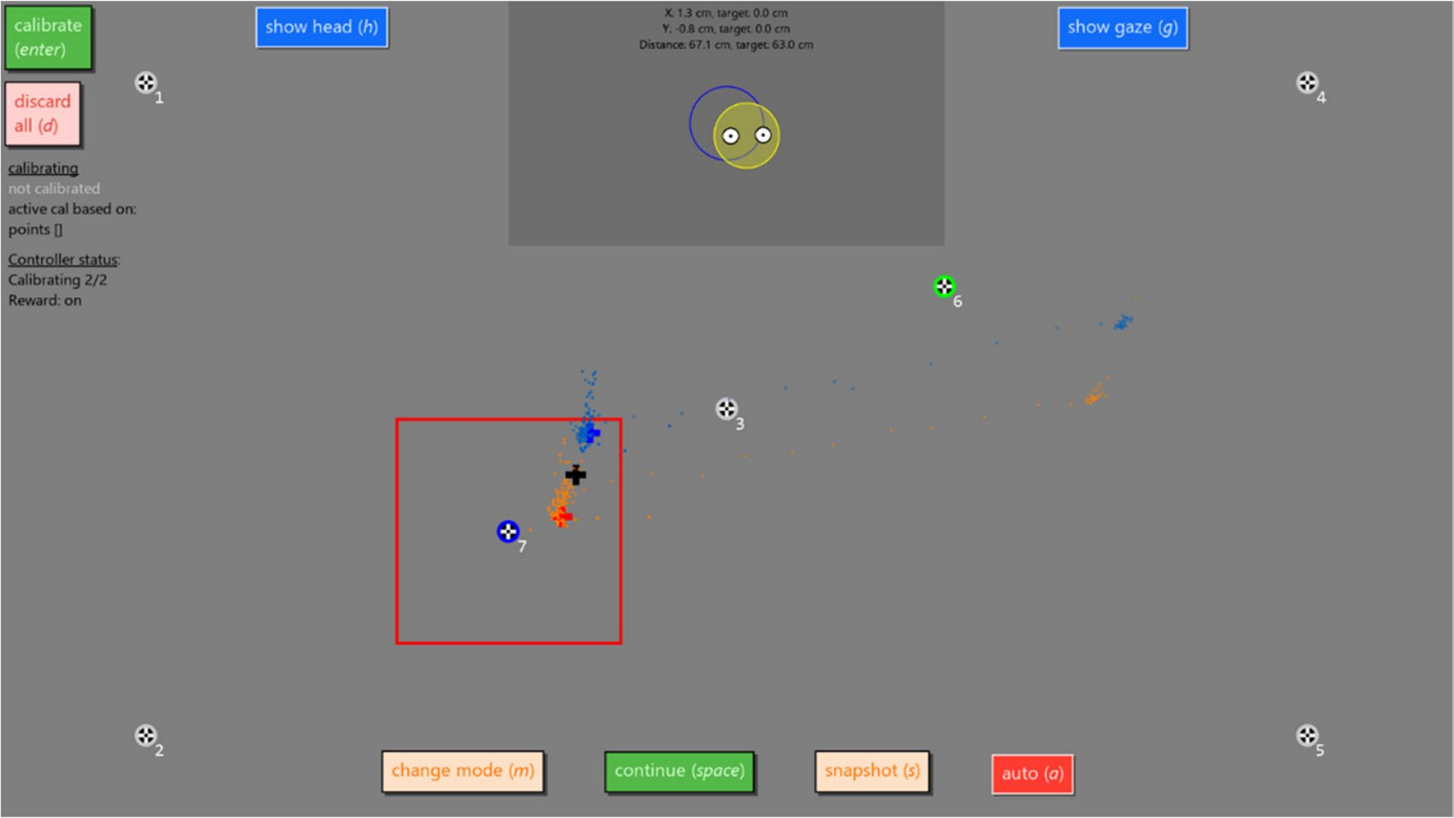
Screenshot of the calibration interface with the NonHumanPrimateCalController calibration controller class activated and calibrating a chimpanzee with a Tobii Pro TX300. This eye tracker does not support monocular calibration and does not provide eye images, hence the “change eye” and “eye images” buttons, as well as the eye images themselves, are not shown. Shown is the state during the calibration data collection stage of the procedure implemented by this controller, and data is being collected for target 7 (as indicated by the dark blue annulus) after calibration data was successfully collected for target 6 (green annulus). Calibration targets 1–5 are not part of the automated procedure, but the operator can manually decide to show these targets if wanted. Further shown is the location and size of the video that is played (red square) and the internal gaze representation of the calibration controller for the left eye (red cross), right eye (blue cross) and the average of the two eyes (black cross)

Once the (small) calibration video size has been reached, the calibration phase starts. During the calibration phase, a small video is played at preconfigured locations on the eye tracker screen (by default, two calibration locations are used for our procedure). The eye tracker data stream is monitored while the video is shown. Once the gaze position (averaged over the left and right eyes) is away from the center of the video by less than one third of the vertical size of the screen for at least 500 ms, the collection of calibration data is triggered for that calibration target. Once data is collected successfully for that target, the video is shown at the next calibration target location, and the same logic for triggering data collection is used. Figure 5 shows the calibration phase of our procedure while data is being collected for the second calibration target. Once calibration data has been collected successfully for all targets (two per default), the procedure requests a calibration to be computed. If this calibration fails, the collected calibration data for all targets is discarded, and new data is collected for the configured targets. If the calibration is successful, the calibration controller switches itself off, and manual inspection and confirmation by the operator is required to continue to the next phase. To maximize calibration accuracy by focusing the participants’ attention toward the same location within each video, we recommend using videos in which the interesting parts of the scenes are concentrated in the center of the video.

**Fig. 6:**
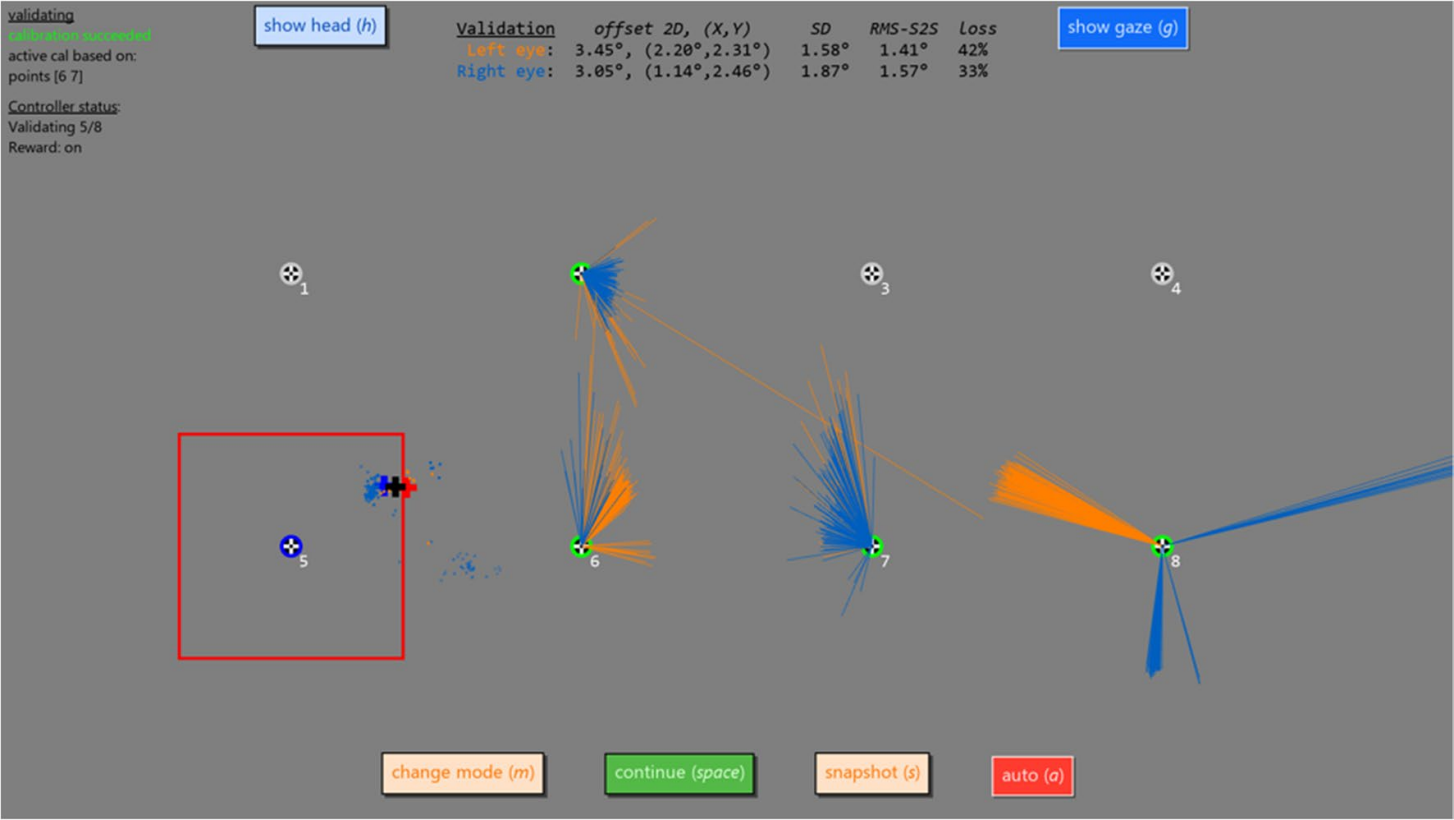
Screenshot of the validation interface with the NonHumanPrimateCalController calibration controller class activated and collecting validation data for a chimpanzee using a Tobii Pro TX300. Depicted is a moment in the validation interval where the controller has collected validation data for four targets (the lines indicate the offset of individual recorded gaze samples from the validation target) while data for the fifth target (number 5, as indicated by the dark blue annulus) is currently being collected. Like in Fig. 5 for the calibration interface, further shown are the location and size of the video that is played (red square) and the internal gaze representation of the calibration controller for the left eye (red cross), right eye (blue cross) and the average of the two eyes (black cross). Information about the quality of the collected validation data, averaged over the validation points for which data is available, is displayed at the top of the screen

The last phase is validation of the calibration to obtain information about the accuracy and other data quality measures for the calibration. The validation phase functions similarly to the calibration phase except that validation data is collected for eight points laid out in a grid of two rows by four columns, and that gaze data had to be on the video instead of the larger spatial limits during calibration for the collection of validation data to be triggered. Small videos are sequentially played at each of the eight validation locations until validation data has been successfully collected for all locations. Figures 6 (chimpanzee) and 7 (cynomolgus macaque) show validation data being collected. During any of the three phases, the experimenter can exit the interface using a keyboard shortcut (shift + escape). For demonstration purposes, we provide a supplemental video (video S1) in which a human participant completes all three phases. Table 1 provides median accuracy and data loss achieved by our automated procedure for a number of chimpanzee, baboon, and cynomolgus macaque participants, providing the reader an indication of what data quality may be expected when working with these participants.

**Fig. 7:**
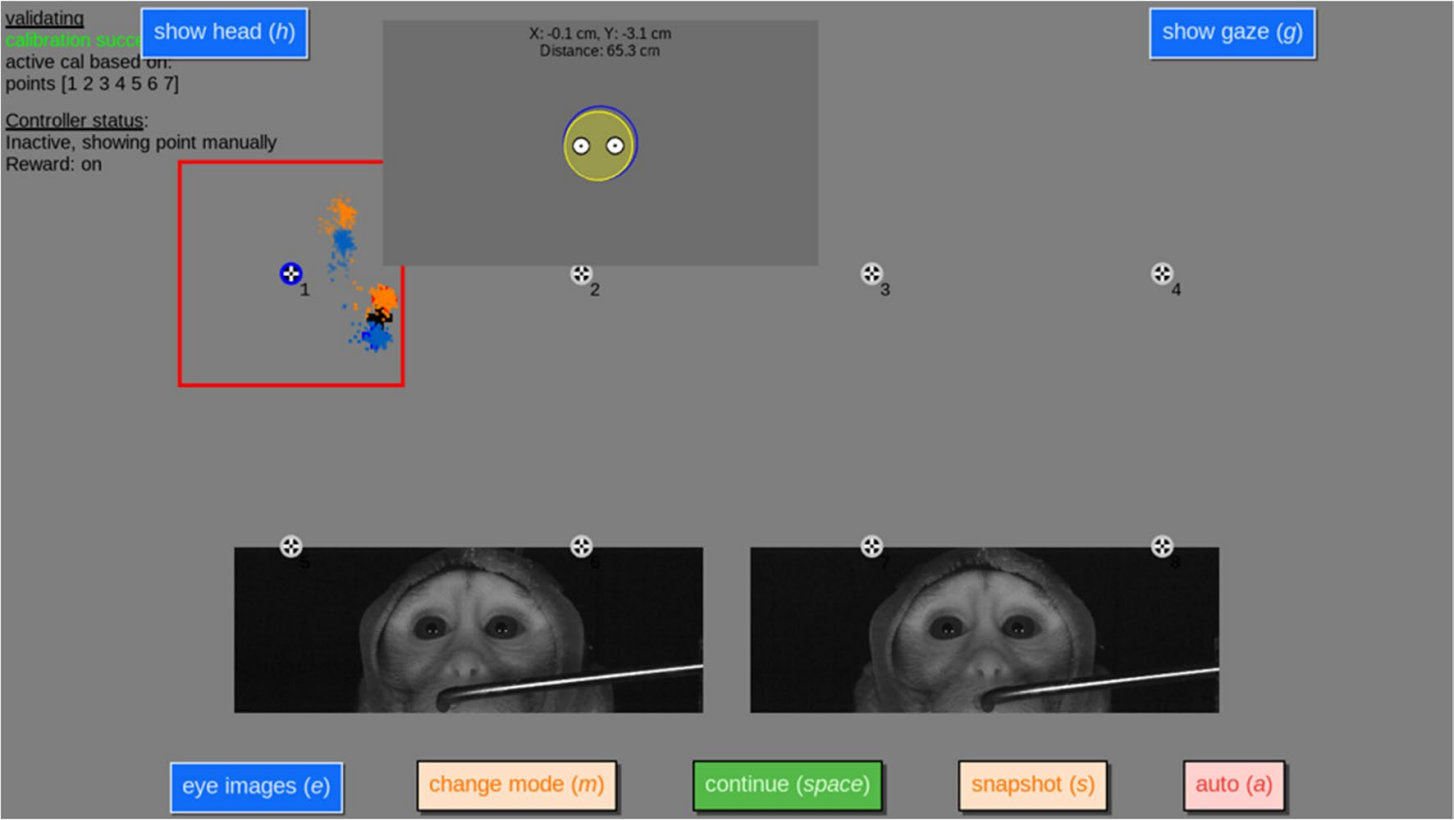
Screenshot of the validation interface with the NonHumanPrimateCalController calibration controller class activated and collecting validation data for a cynomolgus macaque using a Tobii Pro Spectrum. Different from Fig. 6, the operator has manually triggered collection of validation data for the first target. Like in the previous figures, further shown are the location and size of the video that is played (red square), gaze data collected during the last 500 ms (orange and blue dots) and the internal gaze representation of the calibration controller for the left eye (red cross), right eye (blue cross) and the average of the two eyes (black cross). Note: macaques were seated in primate chairs wearing attached lightweight custom 3D-printed helmets (print material: flexible TPU). These are used to maintain a forward-looking posture; and although they do also reduce translational and rotational head movements this is not a requirement for the success of our calibration procedure

**Table 1:** Median accuracy and data loss achieved by our automated procedure for a number of chimpanzee, baboon and cynomolgus macaque participants. Values are based on between three and five sessions per chimpanzee or baboon participant (values within brackets indicate the minimum and maximum observed value) and a single session per cynomolgus macaque participant

	Accuracy (°)			Data loss (%)	
*Eye*	*Left*	*Right*	*Average*	*Left*	*Right*
	**Chimpanzees**				
Ch1	1.71 (1.6–2.1)	2.01 (1.8–2.9)	1.85 (1.8–2.5)	17.2 (9–30)	14.4 (5–36)
Ch2	2.15 (2.0–3.6)	2.20 (1.7–3.1)	2.22 (1.9–3.3)	12.5 (3–14)	03.2 (1–50)
Ch3	2.82 (1.8–3.6)	2.12 (1.8–2.6)	2.72 (1.8–2.9)	20.8 (0–56)	01.8 (0–18)
Ch4	2.01 (1.7–3.0)	2.12 (1.6–2.6)	2.07 (1.7–2.7)	12.1 (4–28)	21.1 (19–36)
Ch5	2.42 (1.9–3.0)	2.75 (1.5–3.8)	2.64 (2.0–3.4)	15.2 (4–29)	25.0 (14–55)
Ch6	2.31 (1.6–2.4)	1.89 (1.7–2.5)	2.10 (1.6–2.5)	27.3 (3–33)	08.2 (0–23)
			*Mean: 2.27*		
	**Baboons**				
B1	1.81 (1.8–2.5)	2.07 (1.5–2.2)	1.93 (1.6–2.3)	14.3 (5–15)	17.6 (4–28)
B2	2.37 (1.9–3.9)	1.88 (1.8–2.3)	2.22 (1.9–2.9)	16.4 (0–41)	03.0 (0–22)
B3	1.82 (1.3–2.1)	2.18 (1.6–3.3)	2.03 (1.4–2.6)	04.5 (2–31)	23.9 (1–54)
B4	1.92 (1.7–2.3)	2.12 (1.7–3.5)	1.99 (1.8–2.9)	03.8 (0–38)	04.8 (0–30)
B5	2.70 (2.1–3.9)	2.70 (2.1–3.0)	2.85 (2.1–3.1)	00.5 (0–70)	14.3 (0–16)
			*Mean: 2.20*		
	**Cynomolgus macaque**				
CM1	0.99	1.15	1.07	05.1	05.1
CM2	0.75	0.69	0.72	03.4	06.8
CM3	1.50	1.41	1.45	10.4	14.0
CM4	1.10	0.99	1.05	11.2	12.4
CM5	1.94	1.65	1.80	05.7	13.0
			*Mean: 1.22*		

## Conclusion

We presented an extension of the Titta toolbox that allows for flexible calibration of participants and standardized validation using desktop Tobii eye trackers. This extension offers additional functionality for existing Titta users as well as a new set of standardized methods appropriate for participants who are unable to follow instructions that are currently underserved by publicly available eye-tracking software environments such as Titta. This toolbox is especially well suited for participants that do not follow verbal instructions, including nonhuman primates, like the baboons, chimpanzees, and cynomolgus macaques described above, and potentially also infants, children, and dogs. An automated calibration procedure for nonhuman primates is included.

## Supplementary information

Below is the link to the electronic supplementary material.

Supplementary file1 Video S1 A demonstration in which a human participant completes all three phases of the automated calibration procedure for nonhuman primates that is implemented by the NonHumanPrimateCalController calibration controller class. A Tobii X2-60 eye tracker was used in this demonstration. This eye tracker does not support monocular calibration and does not provide eye images, hence the “change eye” and “eye images” buttons are not shown in the interface (MOV 98157 KB)

## Data Availability

Not applicable.

## Footnotes

1 It should be noted that it cannot be guaranteed for all eye trackers that the calibration state is cleared upon entering the calibration interface. While the appropriate instructions are sent to the Tobii software development kit, the calibration-clearing functionality appears to not be implemented for all eye trackers. For instance, while it works as expected for a Tobii Pro Spectrum, we have found that calibration state is not cleared for a Tobii Pro TX300. Having an active calibration from a previous participant may make it more difficult to calibrate the next participant due to large offsets in reported gaze position. Shutting off and restarting the eye tracker in this case reliably clears the calibration.

## Code availability

The discussed toolbox is available from https://github.com/dcnieho/titta.

## Declarations

### Open practices statement

The discussed toolbox is available from https://github.com/dcnieho/titta.
